# *microbiomeDASim*: Simulating longitudinal differential abundance for microbiome data

**DOI:** 10.12688/f1000research.20660.2

**Published:** 2020-02-26

**Authors:** Justin Williams, Hector Corrada Bravo, Jennifer Tom, Joseph Nathaniel Paulson

**Affiliations:** 1 Department of Biostatistics, Genentech, Inc, South San Francisco, CA, 94080, USA; 2 Department of Biostatistics, University of California, Los Angeles, Los Angeles, CA, 90095, USA; 3 Department of Computer Science, University of Maryland, College Park, College Park, MD, 24072, USA

**Keywords:** Microbiome, Differential Abundance, Longitudinal, R, Bioconductor

## Abstract

An increasing emphasis on understanding the dynamics of microbial communities in various settings has led to the proliferation of longitudinal metagenomic sampling studies. Data from whole metagenomic shotgun sequencing and marker-gene survey studies have characteristics that drive novel statistical methodological development for estimating time intervals of differential abundance. In designing a study and the frequency of collection prior to a study, one may wish to model the ability to detect an effect, e.g., there may be issues with respect to cost, ease of access, etc. Additionally, while every study is unique, it is possible that in certain scenarios one statistical framework may be more appropriate than another. Here, we present a simulation paradigm implemented in the R Bioconductor software package microbiomeDASim available at http://bioconductor.org/packages/microbiomeDASim microbiomeDASim. microbiomeDASim allows investigators to simulate longitudinal differential abundant microbiome features with a variety of known functional forms with flexible parameters to control desired signal-to-noise ratio. We present metrics of success results on one particular method called metaSplines.

## Introduction

Analysis of the microbiome aims to characterize the composition and functional potential of microbes in a particular ecosystem. Recent studies have shown the gut microbiome plays an important role in various diseases, from the efficacy of cancer immunotherapy to the pathogenesis of inflammatory bowel disease (IBD) ^1– 4^. While many studies profile static community “snapshots”, microbial communities do not exist within an equilibrium ^5^. To better understand bacterial population dynamics, many studies are expanding to longitudinal sampling and foregoing cross-sectional or single time-point explorations. With a decrease in sequencing costs, more longitudinal data will be generated for varying communities of interest. While data generation will present fewer difficulties, there remain several statistical challenges involved in analyzing these datasets.

The common approach in the marker-gene survey literature is to perform pairwise differential abundance tests between specific time points and visually confirm, sometimes using smoothing methods like splines, how differences are manifested across time ^6^. These methods require that analysts provide one or more specific time points to test, and the statistical inferences derived from these procedures are specific to these pairwise tests. Other standard methods for longitudinal analysis test for global differences across time, sometimes using non-linear methods including splines to capture dynamic profiles across time ^7^. Incorporating confounding sources of variability, both biological and technical is essential in high-throughput studies ^8^ and require statistical methods capable of estimating both smooth functions and sample-specific characteristics.

Simulating marker-gene amplicon sequencing data presents a variety of challenges related to biological and technical limitations when collecting data. We present a framework for simulating data that can be used across multiple methods for estimating longitudinal differential abundance. This simulation framework allows for appropriate comparison between methods while taking into account some of the unique challenges for the marker-gene amplicon sequencing data, including the following:

1. Non-negative restriction
2. Presence of Missing Data/High Number of Zero Reads
3. Low Number of Repeated Measurements
4. Asynchronous Repeated Measures
5. Small Number of Subjects

The first two challenges described above are related to the data generating process itself while the following three represent logistical challenges often faced when collecting the data. In microbiomeDASim ^9^, we attempt to address these data generating challenges through specific simulation mechanisms described in the *Microbiome adaptions* section. Similarly, logistical challenges are addressed by allowing users to specify these values flexibly and investigate the corresponding effects, tailoring the simulation to an appropriate setting.

This package allows investigators to simulate longitudinal differentially abundant microbiome features with a variety of known functional forms along with flexible parameters to control design aspects such as signal to noise ratio, correlation structure, and effect size. This feature simulation paradigm can be used in study design evaluation by either matching previously observed trends from small scale studies or evaluating the power to detect differential abundance with a specified study duration, sample size, effect size, effect shape and sample collection schedule. We highlight the ability of the package to use results from a historical longitudinal study on the human gut microbiome in gnotobiotic mice ^10^ to simulate differential abundance for a hypothetical large scale expansion of this study and then demonstrate using the simulation package to evaluate the performance of one particular method of differential abundance estimation across a range of parameter values, metaSplines ^11^.

## Methods

### Distributional assumptions

Sequencing data are often non-normal. However, transformations, such as log(·) or arcsinh(·), are often applied to raw marker-gene amplicon sequencing data so that the subsequent data is approximately normally distributed. As such, we generate simulated data from a multivariate normal distribution. Using a multivariate normal is a natural choice in this setting as longitudinal correlation structure can be easily incorporated. The following methods focus on cases where the desired microbiome features following appropriate transformation are approximately normally distributed.

Assume that we have data generated from the following distribution,

Y∼N(μ,∑),

where

$$\text{Y}=\left(\begin{array}{c} {\text{Y}}_{1}^{T}\\ {\text{Y}}_{2}^{T}\\ \vdots \\ {\text{Y}}_{n}^{T} \end{array}\right)=\left(\begin{array}{c} Y_{11}\\ Y_{12}\\ \vdots \\ Y_{1q_{1}}\\ Y_{21}\\ \vdots \\ Y_{2q_{2}}\\ \vdots \\ Y_{nq_{n}} \end{array}\right),$$

with *Y _ij_* representing the *i ^th^* individual at the *j ^th^* time point and each individual has *q _i_* repeated measurements with *i* ∈ {1, … , *n*} and *j* ∈ {1, … , *q _i_*}. We define the total number of observations as $N=\sum_{i=1}^{n}q_{i}.$ While this model holds for different choices of *q _i_*, throughout this article we will assume, without loss of generality, that the number of repeated measurements is constant, i.e., *q _i_* = *q ∀ i ∈* {1, … , *n*}. This means that the total number of observations simplifies to the expression *N* = *qn*. Similarly, we split the total patients ( *n*) into two groups, control ( *n*_0_) and treatment ( *n*_1_), with the first *n*_0_ patients representing the control patients and the remaining *n–n*_0_ representing the treatment patients. Subsequently we define the total number of observations in each group as *N*_0_ = *n*_0_ · *q* and *N*_1_ = *n*_1_ · *q* respectively. **Y** represents a single taxa/feature to be simulated across the *N* samples. When simulating multiple features as shown later in the gen_norm_microbiome, these features are assumed to be independent.

### Mean components

Partitioning our observations into control and treatment groups in this way allows us to define the mean vector separately for each group as ***µ*** = ( ***µ***_0_, ***µ***_1_) where ***µ***_0_ is an *N*_0_ × 1 vector and ***µ***_1_ is an *N*_1_ × 1 vector. To generate differential abundance the mean for the control group is held constant *µ*_0_ **1**_*n*_0_ × 1_, but allow the mean vector for the treatment group to vary as a function of time *µ*_1 *ij*_( *t*) = *µ*_0_ + *f*( *t _j_*) for *i* = 1, … , *n*_1_ and *j* = 1, … , *q*. The form of *f*( *t _j_*) will dictate the functional form of the differential abundance. Note that if *f*( *t*_1_) = 0, then both groups have equal mean at baseline.

### Polynomial functional forms

We allow *f*( *t _j_*) to be specified using polynomial basis as

$$f\left(t_{j}\right)=\beta_{0}+\beta_{1}t_{j}+\beta_{2}t_{j}^{2}+\cdots +\beta_{p}t_{j}^{p}$$

for a *p* dimensional polynomial. We restrict the allowed polynomials to be either linear, *p*=1, quadratic, *p* = 2, or cubic, *p* = 3. For instance, to define a quadratic polynomial one would specify ***β*** = ( *β*_0_, *β*_1_, *β*_2_) ^*T*^ in the following equation,

$$f\left(t_{j}\right)=\beta_{0}+\beta_{1}t_{j}+\beta_{2}t_{j}^{2}.$$

Again, it is important to note that if ***β*** = **0**, that the treatment group is assumed to have no differentially abundant timepoints. Typically to simulate no differential abundance, a linear trend is chosen with *β*_0_ = *β*_1_ = 0.

### Oscillating functional forms

While polynomial functions are often natural choices for longitudinal trends, interest also lies in exploring other non-smooth, i.e., non-differentiable, types of trends. One such form we refer to as oscillating functional forms. These trends include types that transition from linearly increasing to linear decreasing at a point, or vice versa from linearly decreasing to linear increasing. One of the most well known trends of this type is the absolute value function. To allow for flexible choices in oscillating type trends, we allow for these non differentiable linearly connected trends to repeat forming what we call M and W trends. From a biological perspective we could think of these trends as representing spikes in a particular feature that may occur immediately after a treatment dose is given, but then decays rapidly to baseline levels followed by a similar spike and decay upon repeated dosing. These functional trends are operationalized as

$$\begin{array}{l} f\left(t_{j}\right)=\beta_{0}+\beta_{1}I\left(t_{j}<{\text{IP}}_{1}\right)t_{j}+\left(\beta_{0}+\beta_{1}{\text{IP}}_{1}\right)I\left({\text{IP}}_{1}\leq t_{j}<{\text{IP}}_{2}\right)+\left(\beta_{0}+\beta_{1}{\text{IP}}_{1}\right)I\left(t_{j}\geq {\text{IP}}_{3}\right)\\ \;+\frac{\left(-\beta_{0}-\beta_{1}{\text{IP}}_{1}\right)}{{\text{IP}}_{2}-{\text{IP}}_{1}}I\left({\text{IP}}_{1}\leq t_{j}<{\text{IP}}_{2}\right)\left(t_{j}-{\text{IP}}_{1}\right)\\ \;+\frac{\left(\beta_{0}+\beta_{1}{\text{IP}}_{1}\right)}{{\text{IP}}_{3}-{\text{IP}}_{2}}I\left({\text{IP}}_{2}\leq t_{3}<{\text{IP}}_{2}\right)\left(t_{j}-{\text{IP}}_{2}\right)\\ \;+\frac{\left(-\beta_{0}-\beta_{1}{\text{IP}}_{1}\right)}{t_{q}-{\text{IP}}_{3}}I\left(t_{j}\geq {\text{IP}}_{3}\right)\left(t_{j}-{\text{IP}}_{3}\right), \end{array}$$

where IP _*k*_ for *k* = 1, 2, 3 denotes an inflection point where the linear trend changes from increasing to decreasing or vice versa. Note that for these types of trends that the sign of *β*_1_ determines whether the trend is initially increasing, i.e. M, ( *β*_1_ > 0) or initially decreasing, i.e. W, ( *β*_1_ < 0). By construction, we force the trend line to be exactly zero at IP _2_ and by doing so the trend is specified completely as ***β*** = ( *β*_0_, *β*_1_) ^*T*^ and **IP** = (IP _1_, IP _2_, IP _3_) ^*T*^. An implicit restriction on the functional trend is that IP _3_ ≠ *t _q_*. However, we can construct absolute value and inverted absolute value type trends by defining IP _1_ ∈ ( *t*_1_, *t _q_*) and IP _2_, IP _3_ > *t _q_*. Again, the key difference for these set of trends is that the inflection points create non-smooth trends.

### Hockey stick functional forms

An additional extension to linear functional trends is the family of Hockey Stick functional forms. There are two available families of hockey stick functional forms, which are referred to as L_up and L_down within the package. Both of these trends are designed to create two mutually exclusive regions over the time frame specified. These two regions are defined as *ℜ*_1_ = ( *t*_1_, IP) and *ℜ*_2_ = (IP, *t _q_*) where one of the regions *ℜ*_1_ or *ℜ*_2_ has linear differential abundance while the other has no differential abundance and IP denotes the inflection point. In the case of the L_up trend, *ℜ*_1_ is defined as the non-differentially abundant region and *ℜ*_2_ is a linearly increasing region. We can define the functional form as

$$f\left(t_{j}\right)=\left(-\beta_{1}\times \text{IP}\right)I\left(t_{j}\geq \text{IP}\right)+\beta_{1}I\left(t_{j}\geq \text{IP}\right)t_{j}$$

Note that with this specification that we do not specify the intercept *β*_0_ and instead only need to specify the slope term *β*_1_ and the appropriate point of change. We restrict the slope term to be positive, i.e., *β*_1_ ∈ (0, ∞) to create the "up" trend.

Conversely, the L_down trend assumes that *ℜ*_1_ is a differentially abundant region that begins with the treatment group higher than the control group and then linearly decreases to the region *ℜ*_2_ where there is no differential abundance. We define this functional form as

$$f\left(t_{j}\right)=\beta_{0}I\left(t_{j}<\frac{-\beta_{0}}{\beta_{1}}\right)+\beta_{1}I\left(t_{j}<\frac{-\beta_{0}}{\beta_{1}}\right)t_{j}$$

Note that in this case we do not specify the point of change directly, but rather it is implicitly implied by the choice of *β*_0_ and *β*_1_, i.e. IP = – *β*_0_/ *β*_1_. To ensure that the trend in *ℜ*_1_ is properly specified, we place additional restrictions on the parameters so that *β*_0_ ∈ (0, ∞) and *β*_1_ ∈ (–∞, 0) to ensure the trend is decreasing and check that the choice of *β*_0_ and *β*_1_ are appropriately defined so that IP ∈ ( *t*_1_, *t _q_*).

**Figure 1.**
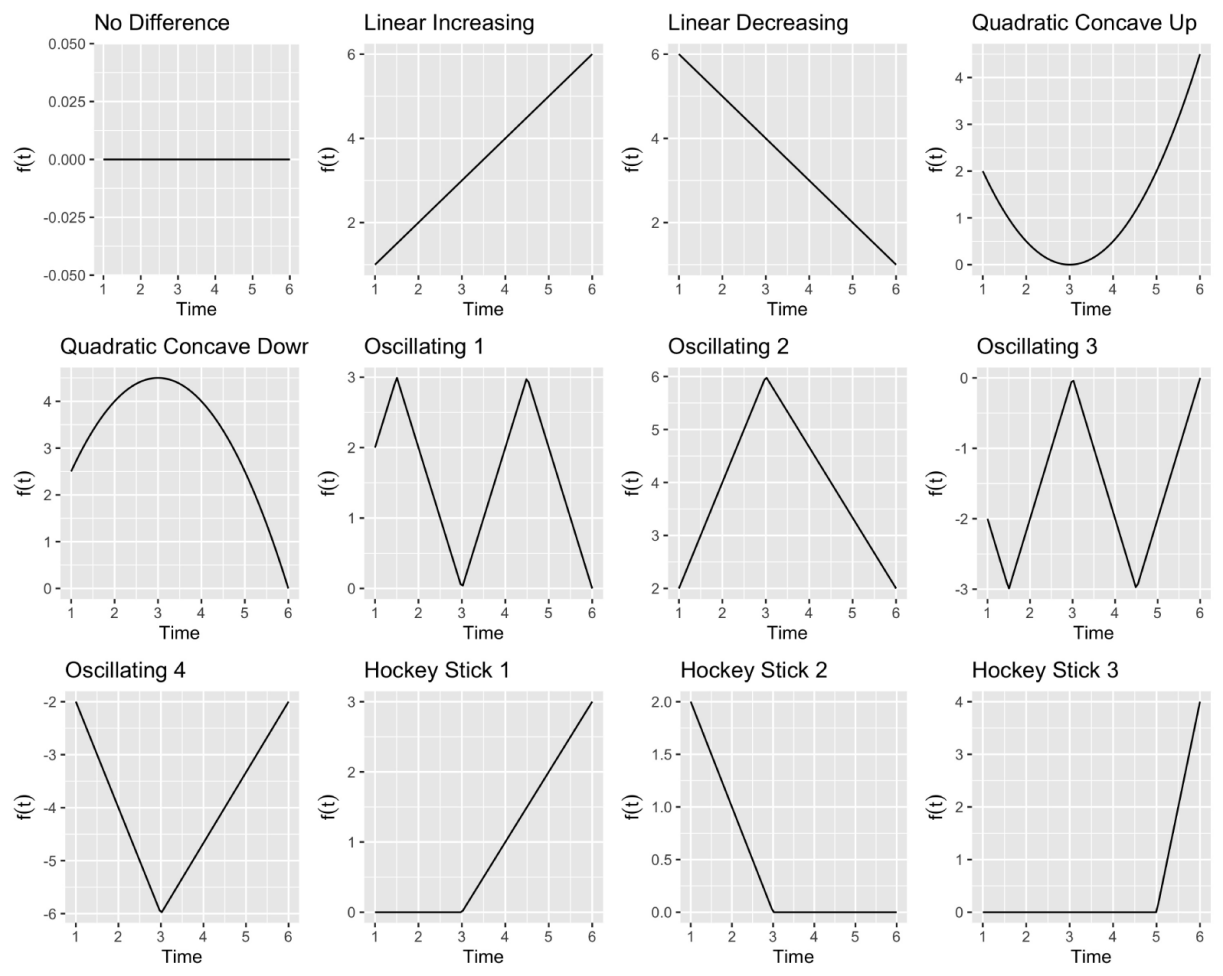
Different functional forms available using the mean_trend() function.

Example trends are shown in Figure 1 generated using the mean_trend function.

### Covariance components

As discussed in the *Introduction*, the multivariate normal is a natural choice for longitudinal simulation due to the ease with which dependency of repeated measures is specified. To encode this longitudinal dependency observations within an individual are assumed to be correlated, i.e. Cor( *Y _ij_*, *Y _ij'_*) ≠ 0 ∀ *j* ≠ *j'* and *i* ∈ {1, … , *n*}, but observations between individuals are assumed independent, i.e. Cor( *Y _ij_*, *Y _i ^'^j_*) = 0 ∀ *i* ≠ *i'* and *j* ∈ {1, … , *q _i_*}. To accomplish this we define the block diagonal matrix **Σ** as **Σ** = bdiag( **Σ**_1_, … , **Σ**_*n*_), where each **Σ** *_i_* is a *q* × *q* covariance matrix for individual *i* and bdiag(·) indicates that the matrix is block diagonal with all off diagonal elements not in **Σ** *_i_* equal to zero. For each individuals covariance matrix, we assume a global standard deviation parameter and correlation component *ρ*, i.e. **Σ** *_i_* = *σ* ^2^ ***Ω***( *ρ*).

For instance, if we want to specify an autoregressive correlation structure for individual *i* the covariance matrix is defined as

$$\sum_{i}=\sigma^{2}\;[\begin{array}{ccccc} 1 & \rho & \rho^{2} & \cdots & \rho^{\left|1-q\right|}\\ \rho & 1 & \rho & \cdots & \rho^{\left|2-q\right|}\\ \rho^{2} & \rho & 1 & & \vdots \\ \vdots & & & \ddots & \vdots \\ \rho^{\left|q-1\right|} & \rho^{\left|q-2\right|} & \cdots & \cdots & 1 \end{array}]$$

In this case we are using the first order autoregressive definition and therefore will refer to this as AR(1).

Alternatively, for the compound correlation structure for an individual *i'* we define the covariance matrix as

$$\sum_{i^{\prime }}=\sigma^{2}\;[\begin{array}{ccccc} 1 & \rho & \rho & \cdots & \rho \\ \rho & 1 & \rho & \cdots & \rho \\ \rho & \rho & 1 & & \vdots \\ \vdots & & & \ddots & \vdots \\ \rho & \rho & \cdots & \cdots & 1 \end{array}]$$

Finally, we allow the user to specify an independent correlation structure for an individual *i''*, which assumes that repeated observations are in fact uncorrelated and is defined as

$$\sum_{i^{\prime \prime }}=\sigma^{2}\;[\begin{array}{ccccc} 1 & 0 & 0 & \cdots & 0\\ 0 & 1 & 0 & \cdots & 0\\ 0 & 0 & 1 & & \vdots \\ \vdots & & & \ddots & \vdots \\ 0 & 0 & \cdots & \cdots & 1 \end{array}]$$

Each of these correlation structures are referred as AR(1), compound, and independent respectively.

### Microbiome adaptions

As discussed in the *Introduction*, simulating microbiome data presents a variety of unique challenges. In particular there are two data generating restrictions, 1. non-negative restriction and 2. presence of missing data/high number of zero reads, that must be addressed when simulating this data. In this section we will outline some of the specific adaptions of the simulation framework designed to address these issues.

***1. Non-negative restriction.*** One of the most relevant challenges faced with microbiome data, is the restriction of the domain to non-negative values. To assure that the simulated normalized counts are non-negative, one solution is to simply replace the multivariate normal distribution with a multivariate *truncated* normal distribution. The new data generating distribution is now

$$\text{Y}∼\text{TN}\left(\mu ,\sum ,a1_{N}\right),$$

where TN indicates the multivariate truncated normal distribution and *a* is the left-truncation value. To impose zero truncation it is assumed that *a* = 0. Values from the multivariate truncated normal are drawn using the package tmvtnorm ^12^. Note that the default method for drawing observations from this distribution is rejection sampling which proceeds by first drawing from a multivariate normal and then for all values that fall below *a* to reject the observed sample and re-sample. This procedure works well when the majority of the distribution falls above the truncation point, but can be computational intensive when the probability of acceptance, *p*_acpt_ = *P*( **Y** > *a* **1**_*N*_), is low. In our simulation design if the value of ***µ*** is sufficiently close to *a* then rejection sampling is not feasible. In the case there the *p*_acpt_ ≤ 0.1, non-negative restriction is imposed by censoring negative values and using point imputation with the truncation value *a* as shown below

$$\begin{array}{l} \;{\text{Y}}^{*}∼N\left(\mu ,\sum \right),\\ Y_{ij}=\{\begin{array}{cc} Y_{ij}^{*} & \text{if}\;Y_{ij}^{*}\geq 0,\\ 0 & \text{if}\;Y_{ij}^{*}<0. \end{array} \end{array}$$

To remove the non-negative restriction there is an option in the function mvrnorm_sim which can be used to turn-off the domain restriction, but by default the zero truncation is imposed. Note that an alternative option to using the multivariate truncated normal is to use the Johnson translation system which can allow samples to be drawn from a multivariate log normal distribution via an appropriate translation function ^13^. The current implementation uses only the multivariate truncated normal distribution for drawing samples via the zero_trunc option within the mvrnorm_sim() and gen_norm_microbiome() functions.

***2. Presence of missing data/high number of zero reads.*** The second major data generating challenge when simulating microbiome data is the presence of missing data along with a high percentage of features with zero counts. Based on technical limitations when amplifying and sequencing microbiome data, certain features may be present but remain undetected. To approximate this potential for missing features that are truly present, options within mvrnorm_sim allow the user to specify: 1) the percent of individuals to generate missing values from ( missing_pct), 2) the number of measurements per individual to assign as missing ( missing_per_subject), and 3) the value to impute for missing observations ( miss_val). Sample IDs are randomly chosen without replacement across all *n* units and for each selected ID measurements are randomly selected without replacement from { *t*_2_ , … , *t _q_*} until the specified number of measurements per individual is achieved. For each missing measurement selected the observed value is replaced with the user specified missing value. Typically the missing value is specified as 0 or as *NA* with the first case representing a situation where the feature was not included due to technical limitations and the second representing an individual whose data was not collected for a particular time point. The initial value *t*_1_ cannot be assigned as missing since it is assumed that all individuals have baseline values collected.

### Implementation

The current version of the R Bioconductor software package microbiomeDASim ^9^ can be installed in R with the following executable code:


if(!requireNamespace("BiocManager", quietly = TRUE)){
   install.packages("BiocManager")
}
BiocManager::install("microbiomeDASim")


Alternatively, a development version is available from GitHub and can be accessed at the following repository williazo/microbiomeDASim. The developmental version may contain additional features that are being developed before they are officially introduced into the Biocondutor version. The developmental version can be installed using the following code:


if(!requireNamespace("devtools", quietly = TRUE)){
   install.packages("devtools")
}
devtools::install_github("williazo/microbiomeDASim")


For a guided introduction into using the functions see either the package vignette for a static example of how to set up and interact with various options for simulating data or for a dynamic guide see mvrnorm_demo.ipynb, a Jupyter notebook on the GitHub page under the inst/script directory. This notebook can be loaded using Google Collab allowing the code to be run without installing Jupyter locally.

### Operation

microbiomeDASim ^9^ is compatible with major operating systems including Mac OS, Windows and Linux. Package dependencies and system requirements are outlined in the documentation available at GitHub.

## Use cases

### Data generating procedure

The primary mechanism for simulating data in the microbiomeDASim package ^9^ is the function mvrnorm_sim. Through this function, the number of subjects in each group is specified along with the necessary parameters, i.e ***β***, *σ* ^2^, *ρ*, and **IP**, to generate ***µ*** and **Σ**. Below is an example of generating differential abundance using a quadratic trend. This type of example could be part of an initial attempt to understand the effects of proposed sample sizes per group, hypothetical functional forms for differential abundance, and sensitivity to signal to noise ratios. In this case there may be a sparsity of empirical evidence and many possible simulation designs can be tested, or on the other end of the spectrum the ecological process could be well understood and the parameter values are well known with emphasis focused on constraints such as collection timepoints and sample size.


> library(microbiomeDASim)
> sim_dt <- mvrnorm_sim(n_control=20, n_treat=20, control_mean=2, sigma=1,
+                       num_timepoints=7, t_interval=c(0, 6), rho=0.7, 
+                       corr_str="compound", func_form="quadratic",
+                       beta=c(0, 3, -0.5), missing_pct=0, missing_per_subject=0,
+                       asynch_time=FALSE, dis_plot=TRUE)
> typeof(sim_dt)
[1] "list"
> names(sim_dt)
[1] "df"        "Y"         "Mu"        "Sigma"     "N"         "miss_data" "Y_obs"
> head(sim_dt$df)
          Y ID time   group     Y_obs
1 0.2132845  1    0 Control 0.2132845
2 0.7784994  1    1 Control 0.7784994
3 1.6464264  1    2 Control 1.6464264
4 1.6283489  1    3 Control 1.6283489
5 0.8769442  1    4 Control 0.8769442
6 0.7625660  1    5 Control 0.7625660
> head(sim_dt$miss_data)
[1] miss_id
<0 rows> (or 0-length row.names)


**Figure 2.**
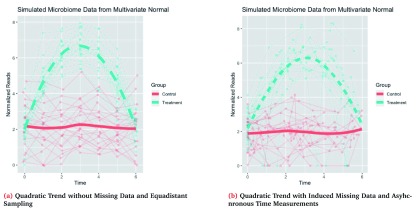
Simulating a quadratic differential abundance trend with compound correlation structure and parameters: *β* = (0, 3, − 0.5) ^*T*^ , *ρ* = 0.7, *σ* = 1, *n*_0_ = *n*_1_ = 20, *q* = 6. Missing data in Figure 2b is generated with 20% of subjects randomly selected to have missing values and for each of these subjects to have 2 non-baseline times randomly selected to be missing with the missing observations imputed as 0.

The output of the simulation function is a list with 7 total objects. The main object of interest is df, which is a data.frame that contains the complete outcome, Y, IDs for each subject *i* = 1, … , *n*, the corresponding time for each observation *t _j_*, a group variable indicator, and the outcome with missing data, Y_obs. The time interval of interest must be specified as a parameter in t_interval, and by default timepoints are drawn at equidistant points along this interval. Both the complete and missing data vectors are also returned as independent objects, Y and Y_obs, respectively, along with the complete mean, ***µ***_*N* × 1_ = Mu, and covariance matrix, **Σ**= Sigma. The function also includes a data.frame miss_data which lists any IDs and time points for which missing data was induced. Finally, the function also returns the total number of observations, N=Σ *_i_ q _i_*. The option dis_plot is used to automatically generate a time-series plot tracking each individuals trajectory along with group mean trajectories. The corresponding plot for this data is shown in Figure 2a.

One important thing to note about the example above is that we generated no missing observations as both missing_pct and missing_per_subject were set to 0. Therefore miss_data was empty. We can compare this to the case below where we induce missingness into the data.


> sim_dt <- mvrnorm_sim(n_control=20, n_treat=20, control_mean=2, sigma=1,
+                       num_timepoints=7, t_interval=c(0, 6), rho=0.7, 
+                       corr_str="compound", func_form="quadratic",
+                       beta=c(0, 3, -0.5), missing_pct=0.2, 
+                       missing_per_subject=2, miss_val=0, asynch_time=TRUE) 
> head(sim_dt$miss_data[order(sim_dt$miss_data$miss_id, sim_dt$miss_data$miss_time),])
   miss_id miss_time
11       6         5
12       6         6
13      13         2
14      13         4
10      16         3
9       16         4
> head(sim_dt$df[sim_dt$df$ID %in% sim_dt$miss_data$miss_id, ])
           Y ID      time   group     Y_obs
36 1.7663067  6 0.0000000 Control 1.7663067
37 0.5918298  6 0.1084354 Control 0.5918298
38 2.0162980  6 1.8372196 Control 2.0162980
39 1.7626451  6 1.8900365 Control 1.7626451
40 2.0873529  6 3.2812129 Control 0.0000000
41 2.1775117  6 5.2906868 Control 0.0000000


In this case we see that for *t*_5_ and *t*_6_ for subject 6 that our outcome with missing data, Y_obs, is now set as 0 which was specified as our missing value while the complete data has the original value before inducing missingness. Another feature demonstrated in this second example is using the asynch_time option. When this variable is set to true, timepoints are randomly drawn from a uniform distribution over the interval [ *t _0_*, *t _q_*]. By construction it is assumed that all individuals have a baseline measurement recorded at t _0_, but all remaining timepoints are drawn at random. The corresponding plot of the outcome Y_obs for this simulation which contains the induced missing observations and asynchronous time measurements is shown in Figure 2b.

As mentioned in the *Distributional assumptions* section, data are generally generated one feature at a time. However, we may want to simultaneously create data with similar patterns across a number of features with certain features experiencing differential abundance while others have no differential abundance patterns. To do this we can use the function gen_norm_microbiome which lets users specify the number of total features to simulate, features, and the number of total features to be differentially abundant, diff_abun_features. In the example below 10 total features are generated with 4 features having longitudinal differential abundance with an L_down hockey stick type trend.


> bug_gen <- gen_norm_microbiome(features=10, diff_abun_features=4, n_control=20,
+                                n_treat=20, control_mean=2, sigma=1,
+                                num_timepoints=5, t_interval= c(0, 10), rho=0.7, 
+                                corr_str="compound",  func_form="L_down", 
+                                beta=c(2, -0.5),  missing_pct=0.2, 
+                                missing_per_subject=2,  miss_val=0)  
Simulating Diff Bugs

  |++++++++++++++++++++++++++++++++++++++++++++++++++| 100% elapsed=05s
Simulating No-Diff Bugs

  |++++++++++++++++++++++++++++++++++++++++++++++++++| 100% elapsed=07s
> head(bug_gen$bug_feat)
         ID time   group Sample_ID
Sample_1  1  0.0 Control  Sample_1
Sample_2  1  2.5 Control  Sample_2
Sample_3  1  5.0 Control  Sample_3
Sample_4  1  7.5 Control  Sample_4
Sample_5  1 10.0 Control  Sample_5
Sample_6  2  0.0 Control  Sample_6
> bug_gen$Y[, 1:5]
              Sample_1 Sample_2 Sample_3  Sample_4     Sample_5
Diff_Bug1   2.78721292 3.034923 2.448909 3.4472145   2.01708421
Diff_Bug2   2.17420076 2.126378 1.875765 2.1224031   1.45393399
Diff_Bug3   3.00420764 2.667490 2.919144 2.5646103   1.98241611
Diff_Bug4   2.79533312 2.526658 2.254584 3.4089330   3.46269243
NoDiffBug_1 1.91089105 2.000122 1.265382 1.1625345   0.97581881
NoDiffBug_2 3.20731129 3.446508 3.389278 3.1057941   4.21898174
NoDiffBug_3 0.05647967 0.000000 1.553321 0.2595184   0.08792209
NoDiffBug_4 2.08900175 1.566923 1.917273 1.4543443   1.34799811
NoDiffBug_5 0.03105152 2.350758 2.139133 1.5934641   0.58829093
NoDiffBug_6 2.24743076 3.082808 2.526052 1.8868046   2.33309314


There are two objects returned in this function, bug_feat and Y. The object bug_feat contains all of the sample specific information including Subject ID, timepoint *t _j_*, an indicator for group assignment and the Sample_ID which ranges from Sample_1 up to Sample_N. The other object Y is the typical OTU (operational taxonomic unit) table with rows corresponding to features and column to samples that are commonly used for analysis in packages such as metagenomeSeq ^14, 15^ and phyloseq ^16^. There are two additional helper functions that will convert the simulated data into MRexperiment or phyloseq objects respectively to allow practitioners to use simulated data in either of these familiar environments.


> # convert to MRexperiment object
> MR_bug_gen <- simulate2MRexperiment(bug_gen)
> MR_bug_gen
MRexperiment (storageMode: environment)
assayData: 10 features, 200 samples
  element names: counts
protocolData: none
phenoData
  sampleNames: Sample_1 Sample_2 ... Sample_200 (200 total)
  varLabels: ID time group Sample_ID
  varMetadata: labelDescription
featureData: none
experimentData: use ’experimentData(object)’
Annotation:
> head(pData(MR_bug_gen))
         ID time   group Sample_ID
Sample_1  1  0.0 Control  Sample_1
Sample_2  1  2.5 Control  Sample_2
Sample_3  1  5.0 Control  Sample_3
Sample_4  1  7.5 Control  Sample_4
Sample_5  1 10.0 Control  Sample_5
Sample_6  2  0.0 Control  Sample_6
>
                            
> # convert to phyloseq object
> phylo_bug_gen <- simulate2phyloseq(bug_gen)
> phylo_bug_gen
phyloseq-class experiment-level object
otu_table()   OTU Table:         [ 10 taxa and 200 samples ]
sample_data() Sample Data:       [ 200 samples by 4 sample variables ]
> head(sample_data(phylo_bug_gen))
Sample Data:        [6 samples by 4 sample variables]:
         ID time   group Sample_ID
Sample_1  1  0.0 Control  Sample_1
Sample_2  1  2.5 Control  Sample_2
Sample_3  1  5.0 Control  Sample_3
Sample_4  1  7.5 Control  Sample_4
Sample_5  1 10.0 Control  Sample_5
Sample_6  2  0.0 Control  Sample_6


### Approximating observed microbiome data

Another important goal of the simulation software is the ability to closely approximate real data from longitudinal experiments where sequencing was performed. To demonstrate this ability using microbiomeDASim we will approximate observed data from a longitudinal study on the human gut microbiome in gnotobiotic mice ^10^. This data file is available within the metagenomeSeq package, and is particularly interesting to simulate for several reasons. The experiment was performed with a total of 12 mice, 6 in each treatment arm, to test the effect of a low-fat, plan polysaccharide-rich diet (BK) versus a high-fat, high-sugar (Western) diet. This small scale study showed promising results and may warrant a larger scale clinical design to investigate the robustness of the effect of diet on the gut microbiome. As such, we can use the simulation tools to generate hypothetical results for this large scale trial assuming that we observe either the same functional trend as the original study or any of the possible hypothetical functional trends at our disposal, including no differential abundance. We will show how to generate hypothetical data for a large scale version of this experiment by increasing the sample size by five fold and replicating the observed functional trend for a particular feature of interest.

**Figure 3.**
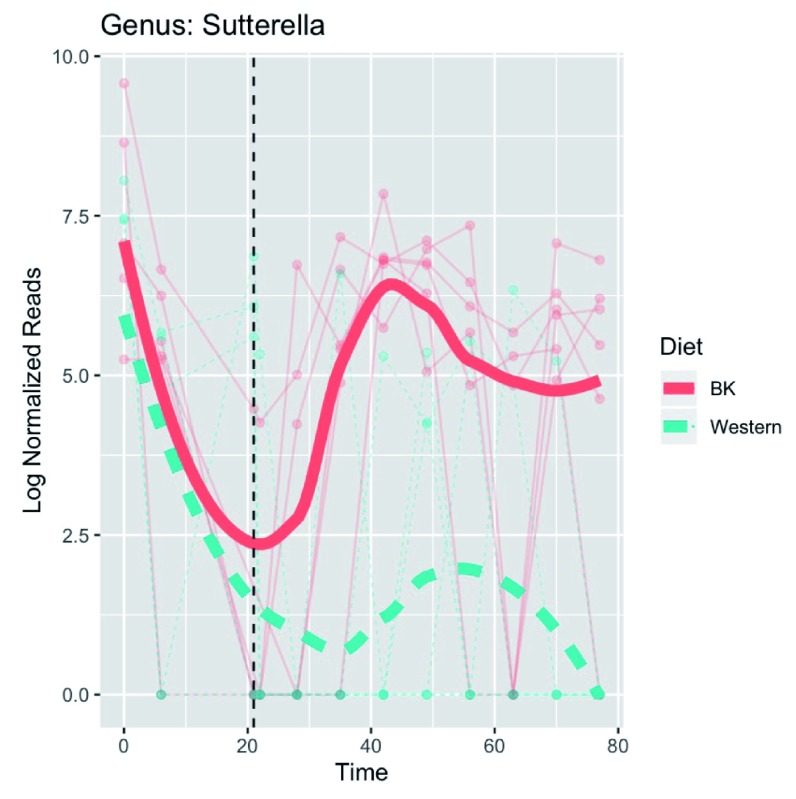
Observed longitudinal trends for the two diet groups in Turnbaugh *et al.* ^10^ study for the Genus *Sutterella* with estimated LOESS curves for each group. Note that both groups had equivalent diets over the first 21 days with half of the mice switching to the Western diet at this point marked with the vertical dotted line.

As a first step we need to identify a particular feature of interest at an appropriate taxonomic level. The original data contains sequenced counts on over 10,000 OTUs with the majority of these being extremely low frequency features. Since the total sample size (n=12) is too small for central limit theory approximations to be valid, we aggregate counts to the genus level for modelling. We further filter genus level features by imposing a minimum depth of 1000 and presence of 10, leaving a set of 35 features. Of these 35 features, we select one at random which we will want to replicate using our simulation framework. In our case we select the genus *Sutterella*. The raw sequencing counts are then log normalized using the default procedure available in the metagenomeSeq package which will serve as our primary outcome of interest. We plot these results over time as shown in Figure 3.

There is significant variability between the groups with a marked decrease in both groups prior to the implementation of the intervention. We see a bounce back effect to baseline levels occurring in the BK diet group while the Western diet group have significantly lower values across the remainder of the study period. We see that the measurement timepoints for each individual vary slightly and are not equally spaced over the entire study window.

**Figure 4.**
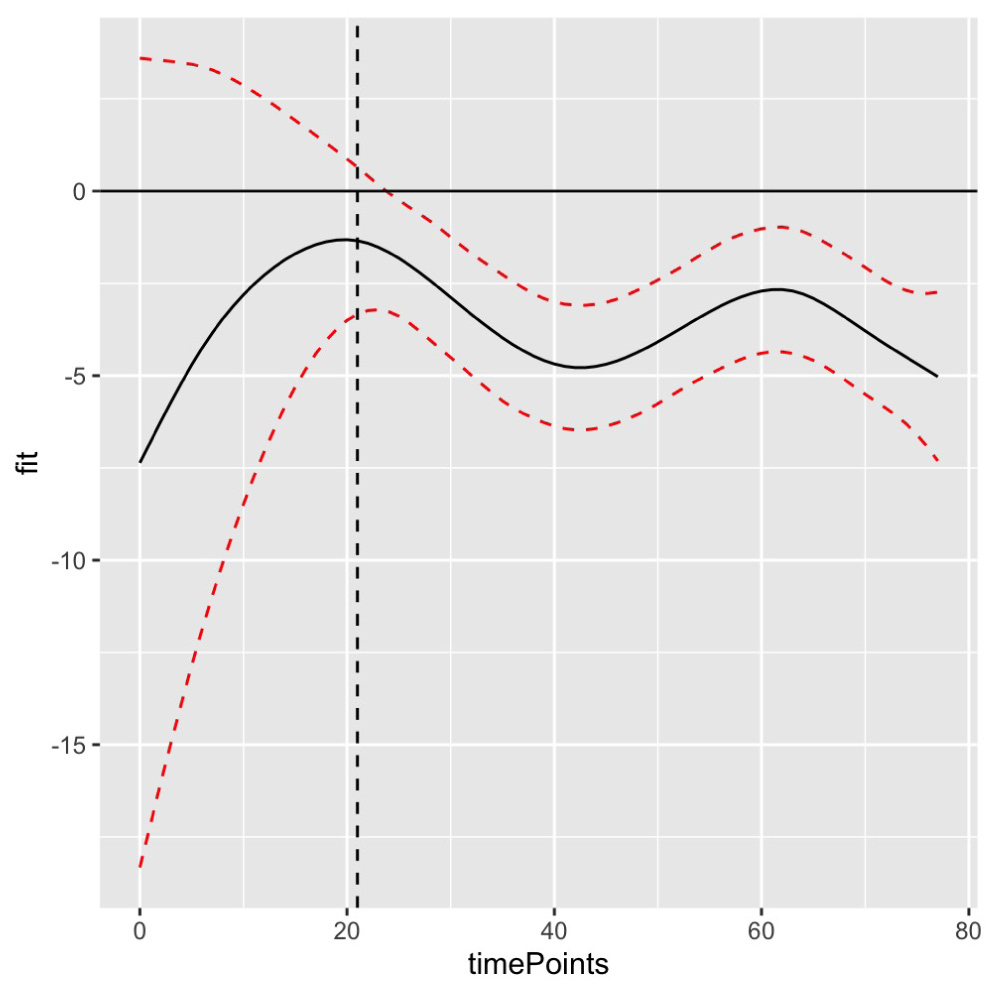
Estimated functional form of the longitudinal differential abundance for the Western diet group from Turnbaugh *et al.* ^10^ study for the Genus *Sutterella*. The black line represents the point estimate with the dashed red lines corresponding to 95% confidence intervals fit using Gaussian smoothed spline ANOVA.

As the primary interest lies in the difference between the diet groups across time, we develop our simulation model by re-scaling the BK reference group to a constant level across time and allowing theWestern group to vary. To obtain an initial estimate of this treatment functional trend we use the metagenomeSeq ^15^ package to fit a Gaussian smoothing spline ANOVA (SS-ANOVA) shown in Figure 4.

**Figure 5.**
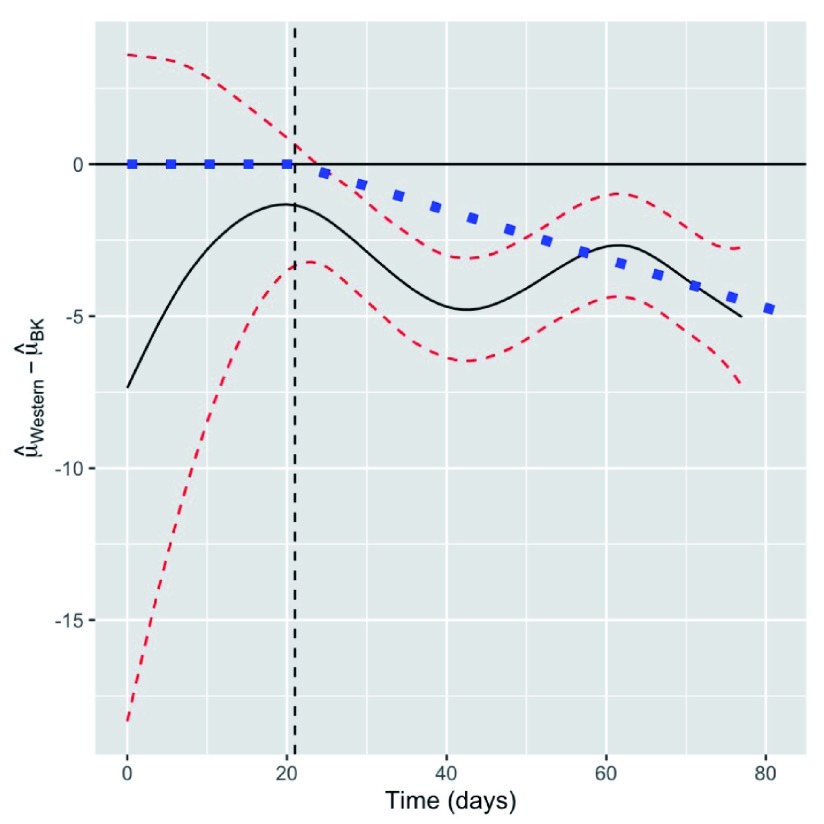
Estimated functional form of the longitudinal differential abundance for the Western diet group from Turnbaugh *et al.* ^10^ study for the Genus *Sutterella* with the corresponding functional form chosen for the simulation shown in blue. The black line represents the point estimate with the dashed red lines corresponding to 95% confidence intervals fit using Gaussian smoothed spline ANOVA.

We see that over the initial 21 days that the 95% confidence intervals for the differential abundance overlaps zero, and that after the intervention begins that the Western group is significantly lower than the BK group. While the estimated trend is non-linear, we may expect that this is a function of small sample size noise and that the true functional trend is a linearly decreasing trend. We therefore construct our hypothetical functional form using the L_up designation assuming there is no differential abundance over the interval *t* ∈ [0, 21] followed by a linearly decreasing trend over the interval *t* ∈ (22,80]. We show this chosen functional form alongside the estimated differential abundance in Figure 5.

In general our hypothetical trend is contained within the estimated bounds of the smoothed fig, and we may believe that it is an ecologically valid representation of the expected change over time.

**Figure 6.**
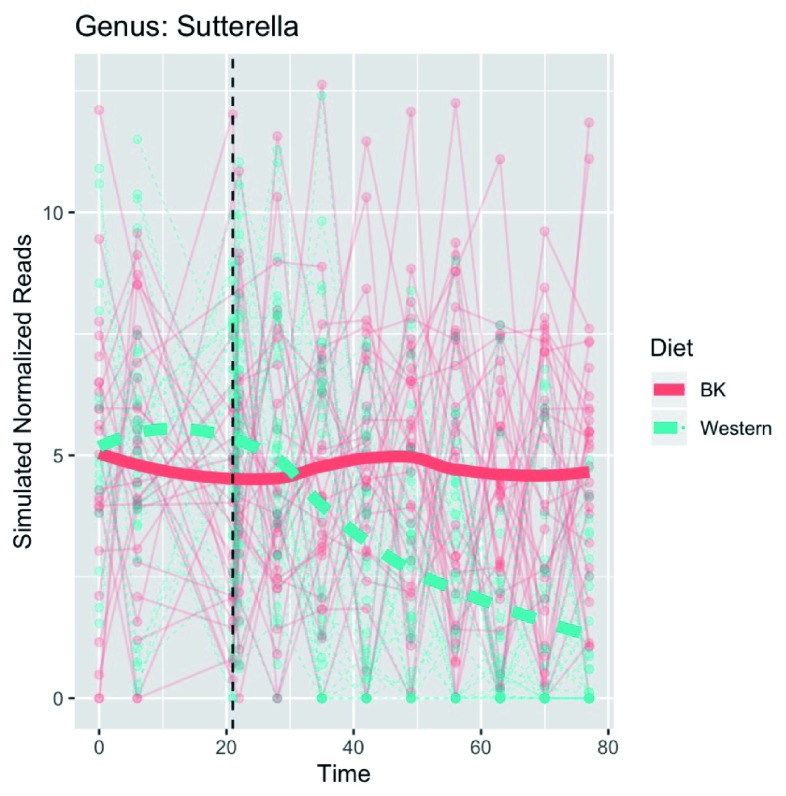
Estimated functional form of the longitudinal differential abundance for the Western diet group from Turnbaugh *et al.* ^10^ study for the Genus *Sutterella* with the corresponding functional form chosen for the simulation shown in blue. The black line represents the point estimate with the dashed red lines corresponding to 95% confidence intervals using metaSplines to fit smoothed spline ANOVA.

With microbiomeDASim we can use the observed times for each ID, and replicate each subject five times creating a total sample size of n=60 with 30 mice in each treatment arm. We use the data to obtain estimates for sigma and control_mean along with the functional form chosen above to generate the simulated data using the mvrnorm_sim_obs() function with an AR1 correlation structure. The results for the simulated data are shown in Figure 6.

This simulated data could then be used to conduct power analyses of detecting differential abundance at time *t* ∈ [ *t*_0_, *t _q_*] or this process could be repeated multiple times to generate feasible bounds for what the trend may look like in this larger sample. Alternatively, the observed data could be altered to change the planned time point measurements to see the effect of collecting fewer samples during the follow-up period. In addition, as mentioned earlier in this section multiple functional forms could be tested including situations where no differential abundance is observed to determine the likelihood of committing Type 1 errors. Further details and code for this example are available on GitHub at inst/script/mouse_microbiome_approximation.pdf

### Longitudinal differential abundance estimation

Next, we want to use our simulation design to test some of the available methods to estimate longitudinal differential abundance. We will examine properties of the estimation method available in the metagenomeSeq ^15^ package to fit a Gaussian smoothing spline ANOVA (SS-ANOVA) model ^11, 17, 18^ referred to here after as the metaSplines method. We start by generating our simulated data. In this example we will fix parameters to have *q* = 10 repeated measurements on each individual with *n*_0_ = *n*_1_ = 30 individuals per arm.


> #generating the simulated data
> out_sim <- mvrnorm_sim(n_control = 30, n_treat = 30, control_mean = 2, sigma = 1,
+                        num_timepoints = 10, t_interval=c(1, 10),
+                        rho = 0.8, corr_str = "compound",
+                        func_form = "L_up", beta = 0.5, missing_pct = 1,
+                        missing_per_subject = 2, IP = 5)
>
> #capturing the true mean values for the specified functional form
> true_mean <- mean_trend(timepoints=seq_len(10), form = "L_up", beta =  0.5, IP = 5)
>
> MR_mvrnorm <- simulate2MRexperiment(out_sim)
> MR_mvrnorm
MRexperiment (storageMode: environment)
assayData: 1 features, 600 samples
  element names: counts
protocolData: none
phenoData
  sampleNames: 1 2 ... 600 (600 total)
  varLabels: ID time group
  varMetadata: labelDescription
featureData: none
experimentData: use ’experimentData(object)’
Annotation:


After generating the simulated data, we fit the model. Note that one can fit either the outcome with the complete data or the outcome with imputed missing data. In this example we use the complete data. To use the induced missing data when creating the MRexperiment object we would set the missing variable in simulate2MRexperiment to TRUE.



>
> #fitting the metaSplines model with random intercept
> metasplines_mod <- fitTimeSeries(obj = MR_mvrnorm, formula = abundance ~ time*class,
+                                   id = "ID", time = "time", class = "group",
+                                   feature = 1, norm = FALSE, log = FALSE, B = 1000,
+                                   random = ~ 1|id)
Loading required namespace: gss
[1] 100
[1] 200
[1] 300
[1] 400
[1] 500
[1] 600
[1] 700
[1] 800
[1] 900
[1] 1000


Now we can display the estimated interval of differential abundance


> metasplines_mod$timeIntervals
     Interval start Interval end     Area       p.value
[1,]              6           10 6.457622   0.000999001


**Figure 7.**
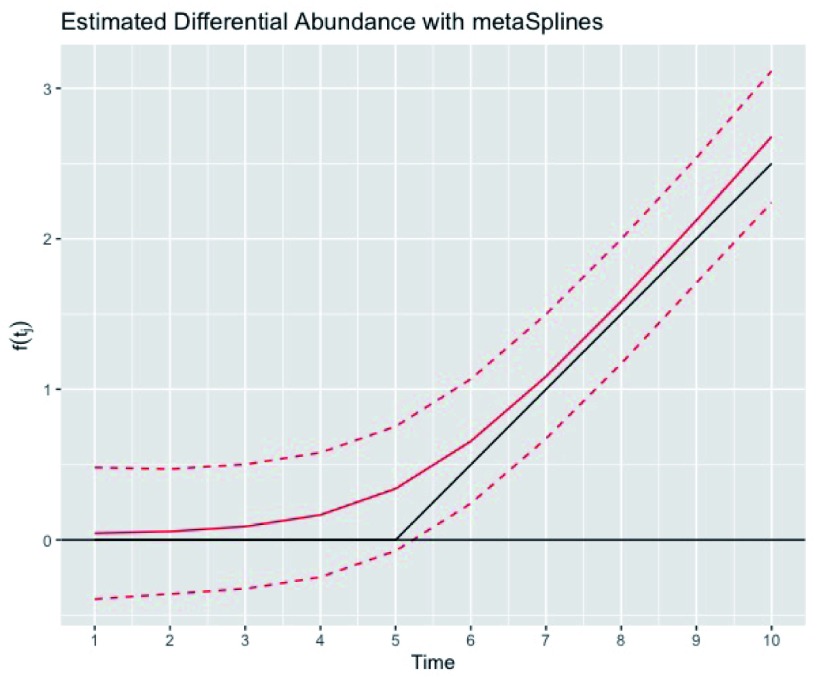
Comparison of the estimated functional form for the metaSplines method, in red, to the truth, in black.

We compare the estimated trend $\hat{f}\left(t_{j}\right)$ to the truth *f*( *t _j_*) as shown in Figure 7. We observe that the metaSplines estimate falls closely to the true functional form. Further, the confidence intervals for the functional form completely contain the true trend reflecting that the variability in estimation is accurately reflected.

### Evaluating estimation procedures

In the example for metaSplines above we looked at performance using a visual inspection for a single choice of parameter values. Using our simulation framework we can expand our investigation of performance. By knowing the true underlying functional form we can quantify how accurate a particular estimation method captures the truth as a function of sample size per group, number of repeated observations, signal-to-noise strength, type of functional form etc. In order to use the simulated data to compare different longitudinal methods for estimating differential abundance we need to define performance metrics that quantify how accurate an estimate is to the truth. We propose four different performance metrics that can be used when comparing methods.

1. Sensitivity/Specificity ∈ [0, 1]
2. Cosine Similarity $\frac{\hat{f}{\left(\text{t}\right)}^{T}f\left(\text{t}\right)}{\|\hat{f}\left(\text{t}\right)\|\cdot \|f\left(\text{t}\right)\|}\in \left[-1,1\right]$
3. Euclidean Distance $\|\hat{f}\left(\text{t}\right)-f\left(\text{t}\right)\|\in \left[0,\infty \right]$
4. Normalized Euclidean Distance $\|\frac{\hat{f}\left(\text{t}\right)}{\|\hat{f}\left(\text{t}\right)\|}-\frac{f\left(\text{t}\right)}{\|f\left(\text{t}\right)\|}\|\in \left[0,2\right]$

To ensure robustness, for each set of parameter values simulated multiple repetitions, *B*, are required. Sensitivity is defined as the number of repetitions where **any** differential abundance at any value *t _j_* ∊ { *t*_1_, . . . , *t _q_*} is detected over the total number of repetitions given that the functional form had some true differential abundance over time, i.e. *f* ( *t _j_*) ≠ 0 ∀ *t _j_* ⇔ ***µ*** **_1_** ≠ ***µ***_**0**_. Likewise, specificity is defined as the number of repetitions where no differential abundance was detected across **all** timepoints over the total number of repetitions given that the function form had no true differential abundance over time, i.e., *f* ( *t _j_*) = 0 ∀ *t _j_*. The other remaining metrics are continuous values that look to compare how closely the estimated mean trend is to the true trend at a set of points *t _j_* ∊ { *t*_1_, . . . , *t _q_*}. Cosine similarity is comparable across different lengths of **t**, but is not particularly discriminant especially near the boundaries around –1 and 1. The Euclidean distance quantifies how far apart each point is but the length of **t** is highly influential. Therefore, to make the Euclidean distance comparable across different lengths of repeated observations we can use the normalized Euclidean distance which first transforms the estimated and true functional form into unit vectors and then calculates the distance between these unit vectors.

### Sensitivity and specificity results

**Figure 8.**
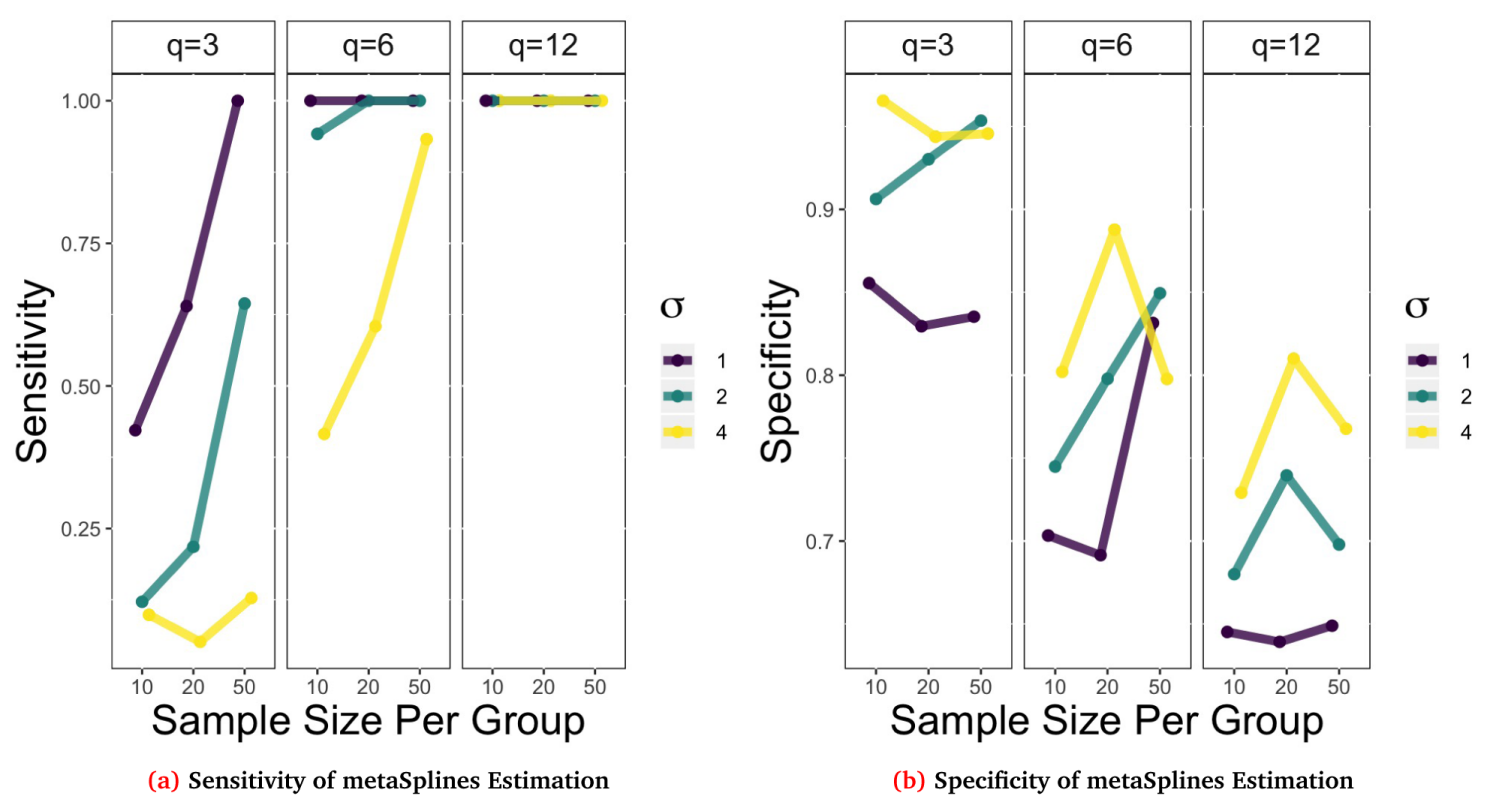
Sensitivity and specificity results for L_up Hockey Stick type trend for an AR(1) correlation structure with parameters: *β* = 1, IP = ( *t _q_* +1)/2, *ρ* = 0.7. Remaining parameters were varied to create 27 different combinations of repeated measurements, sample size per group, and *σ*. Points plot are the average result of *B* = 100 repetitions.

Using these performance metrics we simulated data across a range of different parameters settings and then estimated the functional form of the trend using the metaSplines procedure described earlier for a total of 100 repetitions for each parameter setting. Below we show the performance results for a simulation where the functional form was fixed as L_up with an AR(1) correlation structure, *ρ* = 0.7, and varied the sample size per group, standard deviation, and timepoints from small, medium, and large respectively. The corresponding sensitivity and specificity results are shown in Figure 8a and Figure 8b.

Looking at Figure 8a, in general the sensitivity decreases as *σ* increases for a fixed sample size and *q*. For example when *n*_0_ = *n*_1_ = 10 and *q* = 6 the estimation procedure is perfectly sensitive (100%) when *σ* = 1 but has lower sensitivity (42%) when *σ* = 4. Also as the sample sizes increases for a fixed *q* and *σ*, sensitivity generally increases. Likewise, as the number of repeated observations increase, i.e. *q* increases, the sensitivity increases quite dramatically. This figure suggests that 6 repeated measurements is sufficiently large to detect differential abundance for strong ( *σ* = 1) or medium ( *σ* = 2) signals regardless of the sample size per group. On the other hand, we can look at the specificity in Figure 8b to see that these trends are no longer monotonic. In general we note that as *q* increases the specificity decreases and that as *σ* increases the specificity tends to increase. However, the trend for sample size is more nuanced and may variable due to the number of repetitions that were estimable. Using the metaSplines method there were cases with small sample size and repeated observations that the method returned no estimate.

The sensitivity results shown above were for a single choice of functional form, but this is another potential parameter of interest to test. We ran a similar set of parameter combinations for 7 other functional forms shown in Table 1 below to compare the sensitivity as a function of the type of trend. In this table we can see that the non-differential trends, Oscillating, and variable trends, Hockey Stick, had lower average sensitivity while the linear and quadratic trends tended to perform the best.

**Table 1.** Estimated sensitivity from metaSplines method for data simulated from each respective functional form for a total of 100 repetitions across 27 different parameter settings fixing the correlation structure to be AR(1) with *ρ* = 0.7. Parameter values used: *σ* ∊ {1, 2, 4}, *n*_0_ = *n*_1_ ∊ {10, 20, 50}, *q* ∊ {3, 6, 12}. Note that the Total Non-Missing Observations is less than the Total Observations.

Functional Form	Sensitivity	Total Repetitions	Non-Missing Estimates
Linear Increasing	1.00	2700	2686
Linear Decreasing	0.97	2700	2634
Quadratic: Concave Up	0.91	2700	2154
Quadratic: Concave Down	0.95	2700	2600
Oscillating 1	0.96	2700	2614
Oscillating 2	0.84	2700	2501
Hockey Stick 1	0.78	2700	2261
Hockey Stick 2	0.77	2700	2280

### Continuous performance results

**Figure 9.**
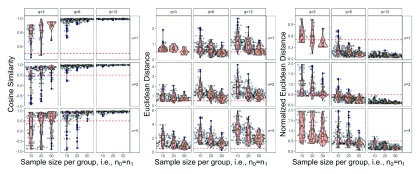
Estimated values of performance metrics including cosine similarity, Euclidean distance, and normalized Euclidean distance based on 100 repetitions for an L_up Hockey Stick trend with AR(1) correlation structure, *ρ* = 0.7, simulated across multiple settings varying repeated measurements *q*, sample size per group, *n*_0_ and *n*_1_ and *σ*. Note that the red dashed line serves as a reference point at 0.5 and the green dot in each panel represents the mean value across the 100 repetitions

The continuous performance metrics for the cosine similarity, Euclidean distance and normalized Euclidean distance are shown in Figure 9 for the L_up trend with AR(1), *ρ* = 0.7. From this figure we see similar trends as the sensitivity results. Starting from the left most panel we see that the cosine similarity is highest when *σ* is small, *q*, *n*_0_, *n*_1_ are large. The spread of cosine similarity scores when *q* = 12 are very tightly clustered around 1 while the spread of values when *q* = 3 or *q* = 6 is larger. The center plot illustrates that using raw Euclidean distances with a small number of repeated measurements tend to have smaller distances, but this trend is not seen with normalized Euclidean distance in the last panel. Within each value of *q* in this middle panel there is a consistent trend that as the sample size per group increases the distance generally decreases. Finally moving to the last panel we have the normalized Euclidean distance, which can now be used to compare across different repeated measurement panels. We see a similar trend to the cosine similarity where the distance decreases, meaning better performance, for small *σ* and large *q* and *n*_0_ = *n*_1_.

Similar to the sensitivity performance metrics shown in Table 1, we can also compare the average value of the continuous performance metrics based on functional form. This is shown in Table 2. Similar trends appear in this table with the linear trends having the highest average cosine similarity scores and lowest average normalized Euclidean distance and non-differentiable trends peforming worse.

**Table 2.** Average continuous performance metrics from metaSplines method for data simulated from each respective functional form for a total of 100 repetitions across 27 different parameter settings fixing the correlation structure to be AR(1) with *ρ* = 0.7. Parameter values used: *σ* ∊ {1, 2, 4}, *n*_0_ = *n*_1_ ∊ {10, 20, 50}, *q* ∊ {3, 6, 12}. Note that the Total Non-Missing Observations is less than the Total Observations.

Functional Form	Total Repetitions	Non-Missing Estimates	Avg. Cosine Similarity	Avg. Euc. Distance	Avg. Norm. Euc. Distance
Linear Increasing	2700	2686	0.99	1.26	0.07
Linear Decreasing	2700	2634	0.98	1.27	0.09
Quadratic: Concave Up	2700	2154	0.94	1.60	0.23
Quadratic: Concave Down	2700	2600	0.97	1.55	0.15
Oscillating 1	2700	2614	0.97	1.69	0.14
Oscillating 2	2700	2501	0.88	1.71	0.35
Hockey Stick 1	2700	2261	0.84	1.35	0.40
Hockey Stick 2	2700	2280	0.84	1.38	0.38

## Conclusions

With an increasing emphasis on understanding the dynamics of microbial communities in various settings, longitudinal sampling studies are underway. There remain many statistical challenges when dealing with longitudinal data collected from marker-gene amplicon sequencing. In order to validate and compare methods of estimation for longitudinal differential abundance a unified simulation framework is needed. Currently available simulation tools include R packages seqtime ^19^ and untb ^20^. These packages focus primarily on simulation from the perspective of ecological processes aimed to capture the entire community dynamics. With microboimeDASim package ^9^ we instead provide the tools to simulate various functional forms for longitudinal differential abundance with added flexibility to control important factors such as the number of repeated measurements per subject, the number of subjects per group, within subject correlation, sequencing of time measurements, etc. for a specific feature of interest. We have shown the benefit of these simulation tools by constructing a simulation design based on real microbiome data and showed the utility in methods evaluation using the metaSplines estimation procedure to compare the performance across a wide range of different parameter settings. In this manner the microbiomeDASim helps meet an important need in the research community to help in study design and compare existing methods as well as validate potentially novel methods.

## Data availability

All data underlying the results are available as part of the article and no additional source data are required.

## Software availability

**microbiomeDASim is available at:** http://bioconductor.org/packages/microbiomeDASim.

**Source code available from:** https://github.com/williazo/microbiomeDASim

**Archived source code at time of publication:** https://doi.org/10.5281/zenodo.3458563 ^9^.

**License:** MIT.
