# Supplementary material for: microbiomeDASim: Simulating longitudinal differential abundance for microbiome data
Source: F1000Res. 2020 Feb 26;8:1769. Originally published 2019 Oct 17. [Version 2] doi: 10.12688/f1000research.20660.2 (PMC7047923; doi:10.12688/f1000research.20660.2)
Supplement: Peer review history

# Peer review: Reviewer response for version 2

**DOI:** 10.5256/f1000research.24837.r60557

**Published:** 2020-02-26

**Authors:** Leo Lahti

**Affiliations:** 1 Department of Future Technologies, University of Turku, Turku, Finland

The authors have responded to my review comments appropriately.

I confirm that I have read this submission and believe that I have an appropriate level of expertise to confirm that it is of an acceptable scientific standard.

# Peer review: Reviewer response for version 1

**DOI:** 10.5256/f1000research.22722.r55802

**Published:** 2019-11-06

**Authors:** Kris Sankaran

**Affiliations:** 1 Montreal Institute for Learning Algorithms (MILA), Montreal, QC, Canada

**Contributions**

 The authors have developed an R package to simulate longitudinal microbiome time course data, especially where there are difference in trajectories between treatment and control groups. This can be used to address,

1. Experimental design: Simulations can guide power analysis, to see whether a proposed study will be well-powered, as a function of assumptions on the generating mechanisms.
2. Methods comparisons: The effectiveness of different methods will depend on the structure of the data, and simulations provide ground truth from which to make assessments.

They simulate data one species at a time. Both treatment and control groups are assumed to have gaussian data, truncated below at 0 to reflect transformed counts. Control data are assumed to be drawn from some common mean, but with specified correlation structure over time. Treatment data are assumed to have a mean that deviates from the control according to some function f(), but have the same correlation structure. The authors provide an interface for simulating a few patterns of f() that are believed to be common in real data (e.g., oscillating, quadratic, and linear shapes).

 The authors share code to display simulated data. They also describe a study evaluating the power of a particular method, 'metaSplines', as simulation parameters are changed.

**Evaluation**

 Strengths:

- I like the idea of formalizing simulation-based power analysis. In the microbiome setting, simulations make more sense than theory, but have two issues (1) they are potentially labor-intensive and (2) they can be ad hoc, and never published. By preparing a package, the authors lower the barrier to entry to / introduce a more formal standard for this work, hopefully enabling simulation-based power analysis in the field.
- The paper is generally technically sound, and reads well. Code is available publicly, is clearly documented, and written in a professional style.

Weaknesses:

- The simulated data are never properly evaluated -- this is my reason for the "partly" response in my report. Of course, any simulation is only an approximation of reality, but it would be nice to know along which dimensions the approximation is close, and along which it is poor. This would also set the stage for studying whether the conclusions that you're aiming for (study design or methods choices) are substantially affected by / robust to these deviations in real data. Something in the spirit of graphical inference could be quite interesting here. ^1^

Missed Opportunities:

- The 'metaSplines' analysis ends somewhat abruptly, because it's not clear what actual conclusions would be drawn from it. I think it would be interesting if you compared another method against it, because you'd be getting at something like the relative efficiency of the approaches (you could also measure their robustness to particular assumptions).
- The functional forms seem somewhat restrictive, though I see their value for people who don't want to spend time writing code. Could you define some kind of interface that makes it easier for people to specify classes of alternatives? E.g., maybe you could let people draw functions interactively, or use as input some examples of microbiome series they see in real data.

**Discussion**

- I have trouble believing in any kind of i.i.d. assumption across species. First, the scale of abundance across species tends to differ by orders of magnitude. Second, many species exhibit very similar behavior.
- Among the controls, couldn't some species also vary over time, because of factors in that individual that change which are not specifically treatment?
- Setting missing data to 0 is generally bad practice, because then you can't distinguish true zeros from missingness. You should either do proper missing data imputation, or recommend methods that explicitly model the missign values / don't require measurements at equal timepoints.
- The different correlation structures you propose reflect an equispaced sampling design. It wouldn't be too hard to change the correlation structure to allow for unevenly spaced sampling, and it would address your point (4, "Asynchronous repeated measures").
- Could you create an interactive notebook? E.g., using binder: https://mybinder.org/v2/gh/krisrs1128/microbiome_dasim_example/master. This would make it easier for people (esp. nonexperts) to get acquainted with your work, without having to install jupyter etc.
- For dosage effects, I'd find a (reversed) sawtooth or wavelet-style spike more believable than an oscillating function. But again, this is related to the point of letting people choose their own alternatives.

**Minor Comments**

- The caption in Figure 5 seems deprecated.
- I don't think you ever defined "OTU".
- The library load should say "microbiome" not "microbime".
- There are still a few typos here and there (e.g., "differential abundant" features and "metrics of success results"), so I recommend another careful read.

I confirm that I have read this submission and believe that I have an appropriate level of expertise to confirm that it is of an acceptable scientific standard.

## References

[1] : Graphical inference for Infovis. *IEEE Trans Vis Comput Graph*.16(6) : 10.1109/TVCG.2010.161 973-9 10.1109/TVCG.2010.161 20975134

# Response

**Published:** 2020-02-19

**Authors:** Justin Williams

**Affiliations:** UCLA Biostatistics, USA

Thank you for your careful review of the manuscript and suggestions. Responses to issues raised are shown below for specific points raised.

**Weakness**

 In the vein of evaluating the robustness of the simulation in approximating reality we have included an additional section “Approximating Observed Microbiome Data” that aims to show how the current package could complement real-world microbiome data. Some of the implications and thought processes for using the simulation package in this setting are discussed within the details of this section. 

 Missed Opportunities

1. We thank the reviewer for this comment. The metaSplines analysis that is included in the manuscript is meant to serve as an illustration of how the simulator could be used to evaluate longitudinal differential abundance methods. In the interest of focusing this software tools manuscript on the simulator package itself, a full comparison of different methods was not investigated. However, this would be a valuable avenue to explore in more depth in a subsequent write-up. 
2. Presently we are not aware of any interface within R that would dynamically allow users to draw functions. This would be highly useful and we would like to continue adding in different functional forms within the package. The currently available forms were an initial foray into some potentially relevant types of trends that might be observed. Users with R expertise can modify the mean_trend function to create alternative functional forms, but allowing full user specification may create an unintended burden for many practitioners. In the future, we will consider some alternative options that allow for higher flexibility while maintaining usability.

**Discussion**

- In our simulation design we are restricting to a single feature of interest when generating data and therefore are inherently ignoring variability across species. This feature simulation can be tailored for individual species of interest and would be run separately in each case.
- The control group could also vary over time, but from a simulation perspective we are treating the design as if the sample has been norm referenced across time for the control group. Since the main goal of estimation is calculating the difference between the treatment and control group over time, restricting the control group to be invariant over time simplifies the user input and maintains the primary goal of estimation.
- By default when inducing missingness in the data, the values are treated as NA rather than 0. However, we included the option to specify the value of the missing data to represent cases where there may be some true non-zero occurrence but due to technical limitations such as read depth the values do not appear. The process of generating missingness is meant to align with some of the typical issues such as loss to follow-up when conducting these types of longitudinal designs.
- Thank you for this comment - as a result we have decided to expand the functionality to allow for asynchronous sampling over a specified interval (using asynch_time=TRUE) or alternatively to have the user specify discrete sampling times for each individual with the mvrnorm_sim_obs function. An example of using each of these asynchronous sampling schemes have been included in the updated manuscript. The compound and independent correlation structures remain unchanged in this unevenly spaced sampling design, but the AR(1) correlation structure now incorporates the amount of time between each sample as |t_{i}-t_{j}|.
- Thank you for this suggestion. The original instructions for installing and running Jupyter with an R kernel were indeed cumbersome. To make the notebook easily interactive, we have re-compiled the materials using Google Colab with a simple badge on top that will allow users to run the code without requiring local installation and setup of Jupyter.
- Thank you for pointing out these possible functional forms. We will work to expand the functional forms available to include these types of trends in the future. As mentioned earlier the ability to define the mean trend has a natural tradeoff between flexibility and useability.

**Minor Comments**

 Caption texts, grammatical errors, and typos pointed out have been corrected. Additional read throughs have also been performed to minimize these types of mistakes in the latest draft.

# Peer review: Reviewer response for version 1

**DOI:** 10.5256/f1000research.22722.r55801

**Published:** 2019-11-05

**Authors:** Leo Lahti

**Affiliations:** 1 Department of Future Technologies, University of Turku, Turku, Finland

This manuscript introduces a new method for simulating longitudinal differential abundance for microbiome data. The method is implemented as an R/Bioc package. The proposed package allows the user to simulate longitudinal microbiome data based on various assumptions, and allows the tuning of key design aspects such as signal-to-noise ratio, correlation structure, effect size and zero inflation. One of the available methods is validated with benchmarking comparisons.

 The manuscript is technically sound and written in a fluent and easily understandable English. Experiments and statistical analyses have been conducted rigorously. The source code and experiments are openly available via Github but I have not tried to replicate the analysis.

 Realistic simulations are valuable for study design, and help to address questions about sample size, density of time points, experimental costs, etc. The work provides pragmatic solutions to a topical problem in microbiome bioinformatics.

**Major comments:**

1. The simulator provides versatile options to tune signal shape, correlations, and noise. However, I am left wondering how well the simulations correspond to real microbiome data. In particular, it is not clear nor validated how the time series shape and correlation structures correspond to known processes in microbial ecology, such as neutral process, competition models (such as generalized Lotka-Volterra), compositionally aware naive models (Dirichlet-Multinomial), mean-reversing processes (Ornstein-Uhlenbeck). All of these have ecological interpretations and have been visible in recent microbiome time series literature. These models are motivated by known ecological processes, rather than technical modifications on the signal shape; it would be relevant to know how large impact the chosen modeling assumptions might have on the results. Can we expect that the proposed simulator will yield qualitative similar conclusions, even if the connection to ecological mechanisms might be weak?
2. The proposed model does not (explicitly) account for heteroschedasticity or overdispersion, and its performance has not been demonstrated with recently popular models of differential abundance, such as DESeq2. It could be true that longitudinal testing of differential abundance requires different methodology. But longitudinal simulators can be also used to simulate cross-sectional data, which is always a snap-shot of longitudinal data. I wonder if the simulator would perform well with standard methods for cross-sectional data; or if it can be shown to yield similar overall distributions. This could provide some additional support for the simulations as the feasibility of the modeling assumptions and their impact on the conclusions remains open.

**Minor comments:**

1. Other simulators for microbiome data and time series are available. One that I am aware of is the seqtime package ( https://github.com/hallucigenia-sparsa/seqtime), although that is only available as an R package (and not formally published), but there may be other recent simulators. I did not find other simulation works being cited, it would be good to check if other simulators can be identified in the recent literature, and how they relate to this work.
2. Lack of integration with phyloseq is a weakness, as this class structure is now very popular among the microbiome R users, and many tools build directly on that class structure. It would be useful addition to the package if the simulations could be made available in a phyloseq format.

I confirm that I have read this submission and believe that I have an appropriate level of expertise to confirm that it is of an acceptable scientific standard, however I have significant reservations, as outlined above.

# Response

**Published:** 2020-02-19

**Authors:** Justin Williams

**Affiliations:** UCLA Biostatistics, USA

Thank you for your review of the manuscript and suggestions for improvement. Both the manuscript and package have been updated to reflect issues raised above. In the following we address point wise specific comments raised from Version 1 of the manuscript.

**Major Comments**:

 (1) Thank you for this comment. As an additional step to address the ability of the simulator to reflect real microbiome data we have provided an example of approximating clinical data with longitudinal microbiome data in mice from Turnbaugh et. al, 2009. This section was added to the manuscript under “Approximating Observed Microbiome Data” with further details about how the simulator can be used to complement and expand clinical efforts. 

 In particular, we outline some of the steps to consider when constructing a simulated dataset to approximate a real-world study. Although our simulation design does not explicitly account for ecological processes as mentioned, the focus on the underlying distributional assumption defines the scope of problems which can be addressed. 

 The simulator looks to construct values for a single feature (aggregated at the taxonomic level of interest) and thus does not incorporate correlation between features or compositional constraints. By focusing on only single features of interest we expect that the simulator will yield similar conclusions to those observed in clinical experiments, and thus offers practitioners a useful tool when designing or expanding a longitudinal microbiome study.

 (2) During the construction of the simulator the variance between both groups is held constant, partly in order to reduce the burden of parameter specification on the user. This choice also reflects a belief that the two groups differ only in their mean trend over time, which is often an appropriate default assumption without particular beliefs about how the heteroskedasticity may differ by group over time. However, it is worthwhile to consider adding a heteroskedastic option to the simulator to incorporate potential differences in noise between groups. While the goal of the simulator focuses on longitudinal designs, it is worthwhile to explore its applicability to cross-sectional data. The simulator function can simulate cross-sectional data by setting num_timepoints=1. Further evaluation of the performance in these cases is merited, but falls outside the scope of this initial software tools manuscript.

**Minor Comments**:

 (1) We thank the reviewer for pointing to these additional simulator packages. A further investigation of the literature returned multiple packages including seqtime, untb, and WrightFisher with similar goals for simulating longitudinal trends. These packages however focus on simulations from a compositional perspective rather than at a single feature level, and lack some of the documentation and formal publication that accompanies our present package. I have updated the manuscript to include references to these additional packages and note some of the differences in the conclusion.

 (2) Thank you for this comment. We have added additional conversion functions simulate2MRexperiment and simulate2phyloseq that format simulated data into the respective objects of interest for the metagenomeSeq and phyloseq packages. We have also added details about using these functions within the manuscript.
